# Pharyngoesophageal reconstruction after resection of hypopharyngeal carcinoma: a new algorithm after analysis of 142 cases

**DOI:** 10.1186/1477-7819-12-182

**Published:** 2014-06-09

**Authors:** Adel Denewer, Ashraf Khater, Mohamed T Hafez, Osama Hussein, Sameh Roshdy, Fayez Shahatto, Waleed Elnahas, Sherif Kotb, Khaled Mowafy

**Affiliations:** 1 Department of Surgical Oncology, Oncology Center (OCMU), Mansoura, Daqahlia, Egypt; 2 Vascular Surgery, Mansoura University, Mansoura, Egypt

## Abstract

**Background:**

The aim of this study is to define an algorithm for the choice of reconstructive method for defects after laryngo-pharyngo-esophagectomy for hypopharyngeal carcinoma.

**Methods:**

One hundred and forty two cases of hypopharyngeal carcinoma were included and operated on by either partial pharyngectomy, total pharyngectomy or esophagectomy. The reconstructive method was tailored according to the resected segment.

**Results:**

Pectoralis flap was used in 48 cases, free jejunal flap in 28 cases, augmented colon bypass in 4 cases, gastric pull up in 32 cases and gastric tube in 30 cases. Mean hospital stay was 12 days. Mortality rate was 10.6% and morbidity rate was 31.7%. Total flap failure occurred in 3 cases of free flap and one case of pectoralis flap. There were 23 cases of early fistula. Late stricture occurred in 19 cases, being highest with myocutaneous flap (early fistula 12/50 and late stricture 13/50).

**Conclusion:**

Free jejunal flap was the flap of choice for reconstruction when the safety margin is still above the clavicle. In cases with added esophagectomy, we recommend gastric tube as a method of choice for reconstruction.

## Background

Hypopharyngeal carcinoma represents a clinical challenge for most surgeons. Most of the patients are diagnosed with a relatively late stage carcinoma, with commonly associated comorbidities and a high rate of regional and distant metastasis [1]. In most series, 70% to 85% of patients have stage III or IV disease at presentation, with 5-year overall survival of 15% to 45% [2]. A multidisciplinary approach is crucial to achieve a more favorable outcome both oncologically and functionally [3]. Poor survival was usually correlated with infiltrated margins, local recurrence or reconstruction failure and perioperative morbidity [4]. In spite of the best efforts to simplify and improve the reconstruction techniques, the complication rates are still relatively high [5]. Many studies declared their methods for reconstruction [6-10], but there were no sharp guidelines for the choice of method tailored for each defect.

### Aims

The aim of this study is to define an algorithm for the choice of method for defect reconstruction after pharyngolaryngectomy for hypopharyngeal carcinoma with the best functional and oncologic results.

## Methods

**Figure 1:**
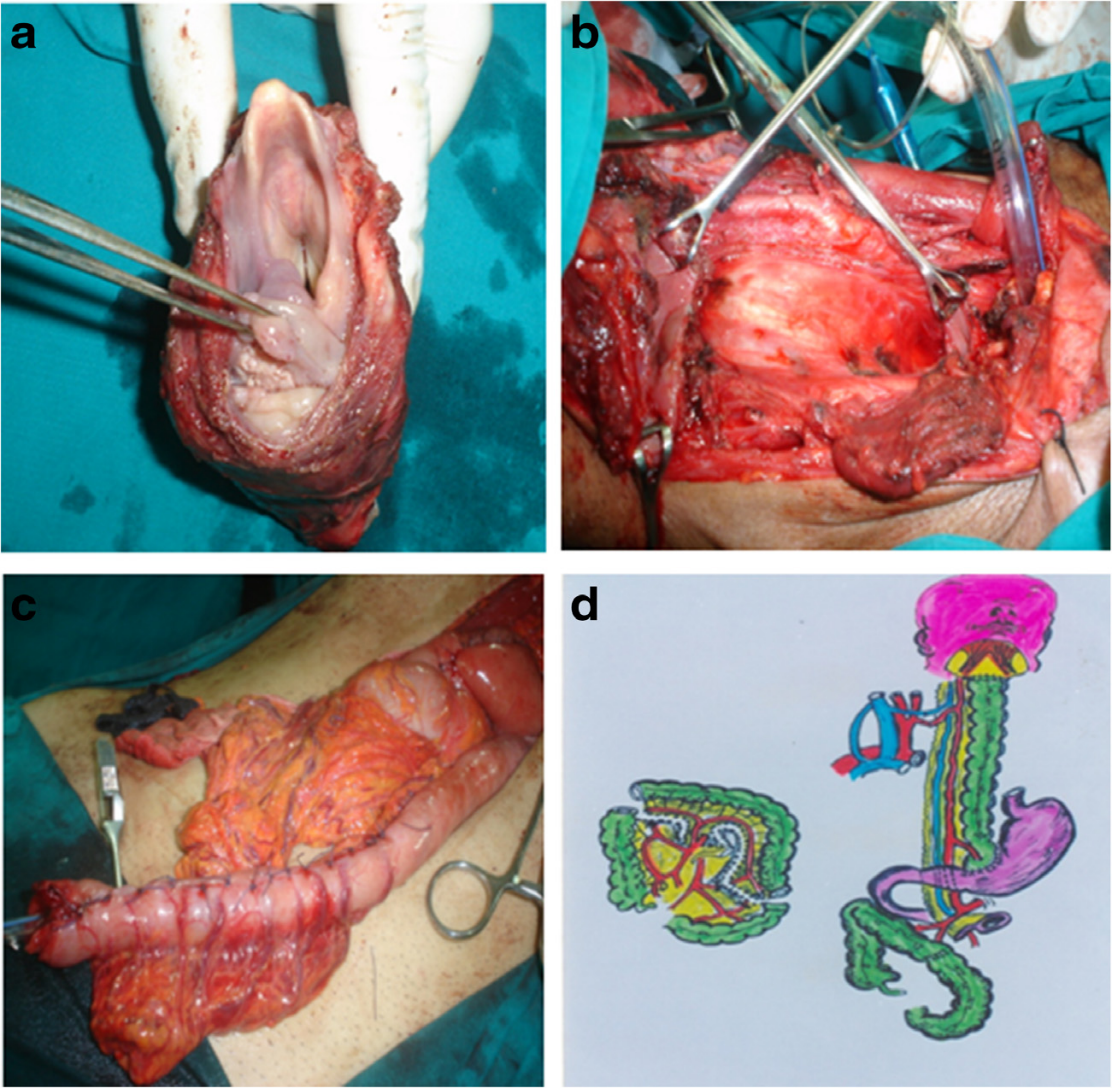
**Operative steps and schematic diagram. (a)** Specimen after removal. **(b)** Empty neck after specimen removal. **(c)** Reversed gastric tube ready for anastomosis in the neck. **(d)** Schematic view to the augmented colon by pass.

**Figure 2:**
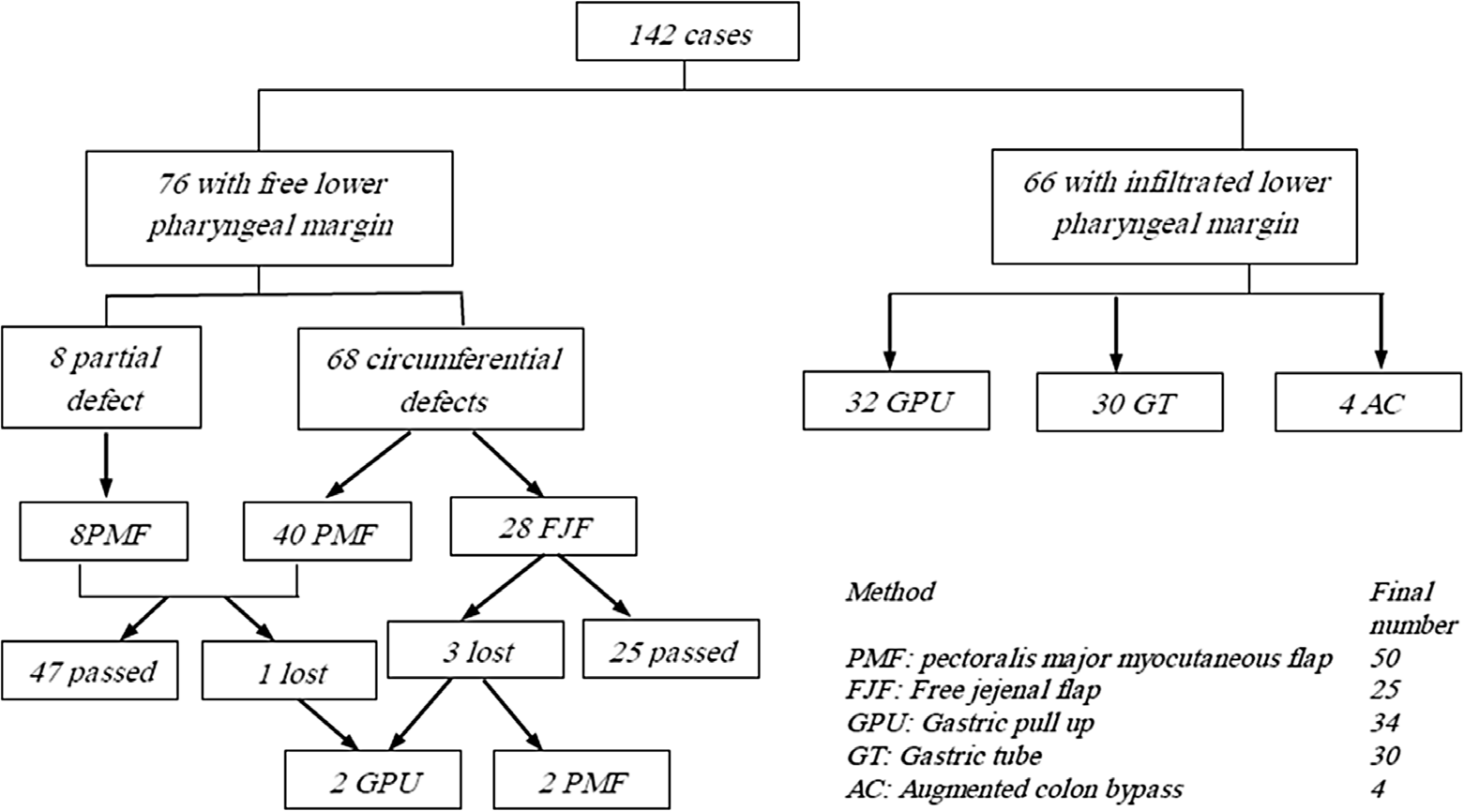
Reconstruction techniques used.

**Figure 3:**
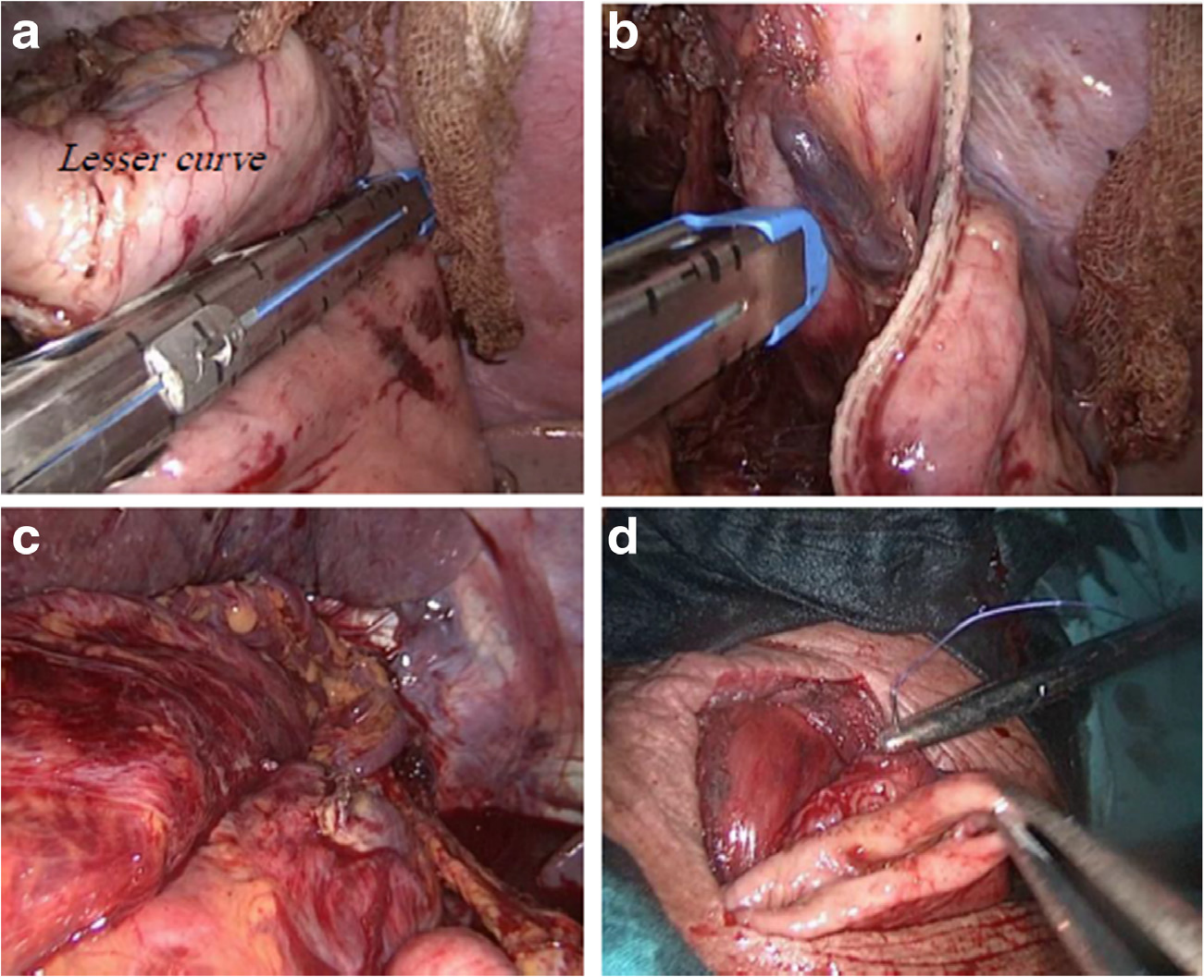
**Reconstruction using laparoscopic gastric tube. (a,b)** Creation of the gastric tube using the Endo-GLA. **(c)** Delivery of the gastric tube to the neck. **(d)** Anastomosis of the tube to the pharynx after dividing the esophageal attachment.

This is a prospective study that was carried out from January 2004 to December 2012 in Mansoura Oncology Center, Mansoura, Egypt. It included 145 patients with pathologically diagnosed hypopharyngeal carcinoma who were candidates for surgical resection. Table 1 shows the demographic data. Patients with carotid artery infiltration, those with metastatic disease, and unfit patients (who cannot tolerate major operation because of associated comorbidity) were excluded from this study. Neoadjuvant chemo-radiotherapy was administered to 80 patients in whom the primary tumor was bulky. In these, there were no cases that showed complete response; partial response occurred in 74 patients and progressive disease was noticed in 6 patients. In all cases surgery was possible except in three patients who developed distant metastases during the neoadjuvant treatment course and they were excluded from the study. Table 2 shows the detailed operative data of the 142 cases. The choice of the operative resection and the method of reconstruction was reviewed by our local institutional board. Partial pharyngectomy was performed in only eight cases, and total pharyngectomy was performed in the rest of the cases. Ipsilateral nodal dissection was carried out in 30 cases, and bilateral nodal dissection was carried out in 72 cases. In 76 cases the lower pharyngeal margin was free. In 8 of them the wall defect was partial and was closed by pectoralis major myocutaneous flap. In the remaining 68 cases the defect was circumferential and was replaced by pectoralis major myocutaneous flap in 40 cases and free jejunal flap in 28 cases (Figure 1). Three cases of the free jejunal flap were totally lost and pectoralis major myocutaneous flap was used as salvage in 2 cases and gastric pull up was used in the third case with success in all. In 66 cases the lower pharyngeal margin was infiltrated and further esophagectomy was performed. In 32 cases gastric pull up was used as a replacement with anastomosis to the upper pharyngeal end in the neck. In 30 cases gastric tube was used (laparoscopic in 8 cases and open in the rest) and in 4 cases augmented colon was used (Figure 2). All reconstructive maneuvers are described in detail in many publications [11-17], and we will highlight only the laparoscopically harvested gastric tube. After completing the pharyngolaryngectomy, the upper esophagus is dissected by blunt dissection until the carina, then abdominal ports are placed in the upper abdomen ensuring that all instruments can reach the level of Louis angle (Figure 3). A liver retractor is applied through the right flank port while two supra-umbilical and two left hypochondrial ports are used as working ports. The greater curve of the stomach is mobilized with the aid of harmonic scalpel by dividing the short gastric and omental vessels (Figure 3). Then the lesser curve was mobilized from the pylorus till the esophageal hiatus by dividing the lesser omentum, preserving the right gastric artery and dividing the left gastric vessels at their origin (Figure 3). Kocherization of the duodenum is performed with mobilization of the duodenum towards the left side. Then the esophageal hiatus is mobilized by dividing the phreno-esophageal ligament, both vagi and the esophageal vessels, and then continuing mediastinal dissection through the hiatus by combined blunt and sharp dissection (Figure 3).

**Table 1:** Patient demographic data

	
Mean age in years (range)	55 (35–67)
Gender	Total 145
Male	117
Female	28
Associated comorbidity	
Diabetes	43
Hypertension	84
Cardiac	25
Chronic obstructive pulmonary disease	64
Chronic liver disease	35
Tumor pathology	
Squamous cell carcinoma	132
Undifferentiated carcinoma	13
Primary tumor site	
Larynx	12
Hypopharynx	133
T stage	
T3	94
T4a	51
N stage	
N0	22
N1	35
N2	85
Neoadjuvant therapy	80

Values are shown as number, unless otherwise indicated.

**Table 2:** Operative details

	
Mean operative time in minutes (range)	360.5 (270–540)
Mean blood loss in ml (range)	270 (230–450)
Type of resection	
Partial pharyngectomy	8
Total pharyngectomy	134
Lymph node dissection	
Ipsilateral	30
bilateral	72
Type of reconstruction	
Myocutaneous flap	48
Free jejunal flap	28
Augmented colon bypass	4
Gastric pull up	32
Gastric tube	30
Mean hospital stay in days (range)	12 (9–20)
Mortality rate	10.6% (15 cases)
Morbidity rate	31.7% (45 cases)

Values are shown as number, unless otherwise indicated.

## Results

This study included 145 cases; 12 cases were of laryngeal origin and 133 cases were of hypopharyngeal origin. Table 1 shows the demographic data of patients. Most of the cases were squamous cell carcinoma (132 cases), and 80 cases received neoadjuvant chemoradiotherapy. During treatment, three cases developed distant metastases and were excluded from the study. For descriptive statistics of quantitative variables the mean, range and standard deviation were used to describe central tendency and dispersion. Survival and disease-free survival analyses were calculated by the Kaplan-Meier Product-Limit Estimator. Median follow-up was 60 months. The disease-free survival rate was 62% by the end of the first year and 25% by the end of the fifth year. The overall survival rate by the end of the first year was 80.5% and 50.7% by the end of the fifth year. Table 2 shows the operative details. The mean operative time was 360.5 minutes. The mean blood loss was 270 ml. Partial pharyngectomy was performed in eight patients while total pharyngectomy was performed in 134 patients; 30 patients underwent ipsilateral block dissection and 72 underwent bilateral block dissection. Eighteen lymph nodes were removed without planned block neck dissection in the other cases. Details about the reconstructive techniques are shown in Figures 2 and 1. The mean hospital stay was 12 days (range 9-20). The mortality rate was 10.6% (15 cases) and the morbidity rate was 31.7% (45 cases). The leading causes of mortality in our series were respiratory complications, sepsis syndrome, and cardiopulmonary complications. Systemic complications are shown in Table 3. There were four cases of total flap failure; three of them were with free jejunal flap (10.7%) and one with pectoralis major myocutaneous flap (2.1%). Early fistula was seen in 12 out of 50 cases of pectoralis major myocutaneous flap (24%), 2 out of 25 cases of free jejunal flap (8%), 1 out of 4 cases with augmented colon bypass (25%), 5 out of 34 cases of gastric pull up (14.7%) ,and 3 out of 30 cases with gastric tube (10%). Late stricture was seen in 13 out of 50 cases with pectoralis major myocutaneous flap (26%), 1 out of 25 cases with free jejunal flap (4%), 3 out of 34 cases with gastric pull up (8.8%), and 2 out of 30 cases with gastric tube (6.7%); there was no recorded stricture with augmented colon bypass. Twenty out of 50 cases (40%) with pectoralis major myocutaneous flap could resume a solid diet; this occurred in 20 out of 25 cases (80%) with free jejunal flap, 3 out of 4 cases (75%) of augmented colon bypass, 28 out of 34 cases (82.4%) with gastric pull up, and 26 out of 30 cases (86.7%) with gastric tube.

**Table 3:** Postoperative complications and functional outcome

	
**Systemic complications**	
Respiratory	15
Cardiac	10
Sepsis syndrome	15
Others	5
Total flap loss	
Free jejunal flap	3 (10.7%)
Pectoralis myocutaneous flap	1 (2.1%)
Early fistula	
Myocutaneous flap	12 out of 50 cases (24%)
Free jejunal flap	2 out of 25 cases (8%)
Augmented colon bypass	1 out of 4 cases (25%)
Gastric pull up	5 out of 34 cases (14.7%)
Gastric tube	3 out of 30 cases (10%)
Late stricture	
Myocutaneous flap	13 out of 50 cases (26%)
Free jejunal flap	1 out of 25 cases (4%)
Augmented colon bypass	0
Gastric pull up	3 out of 34 cases (8.8%)
Gastric tube	2out of 30 cases (6.7%)
Resuming solid diet	
Myocutaneous flap	20 out of 50 cases (40%)
Free jejunal flap	20 out of 25 cases (80%)
Augmented colon bypass	3 out of 4 cases (75%)
Gastric pull up	28 out of 34 cases (82.4%)
Gastric tube	26 out of 30 cases (86.7%)
Median follow-up in months (range)	60 (9–108)
Disease-free survival rates	
1 year	62%
5 year	25%
Overall survival rate	
1 year	80.5%
5 year	50.7%

Values are shown as number, unless otherwise indicated.

## Discussion

**Figure 4:**
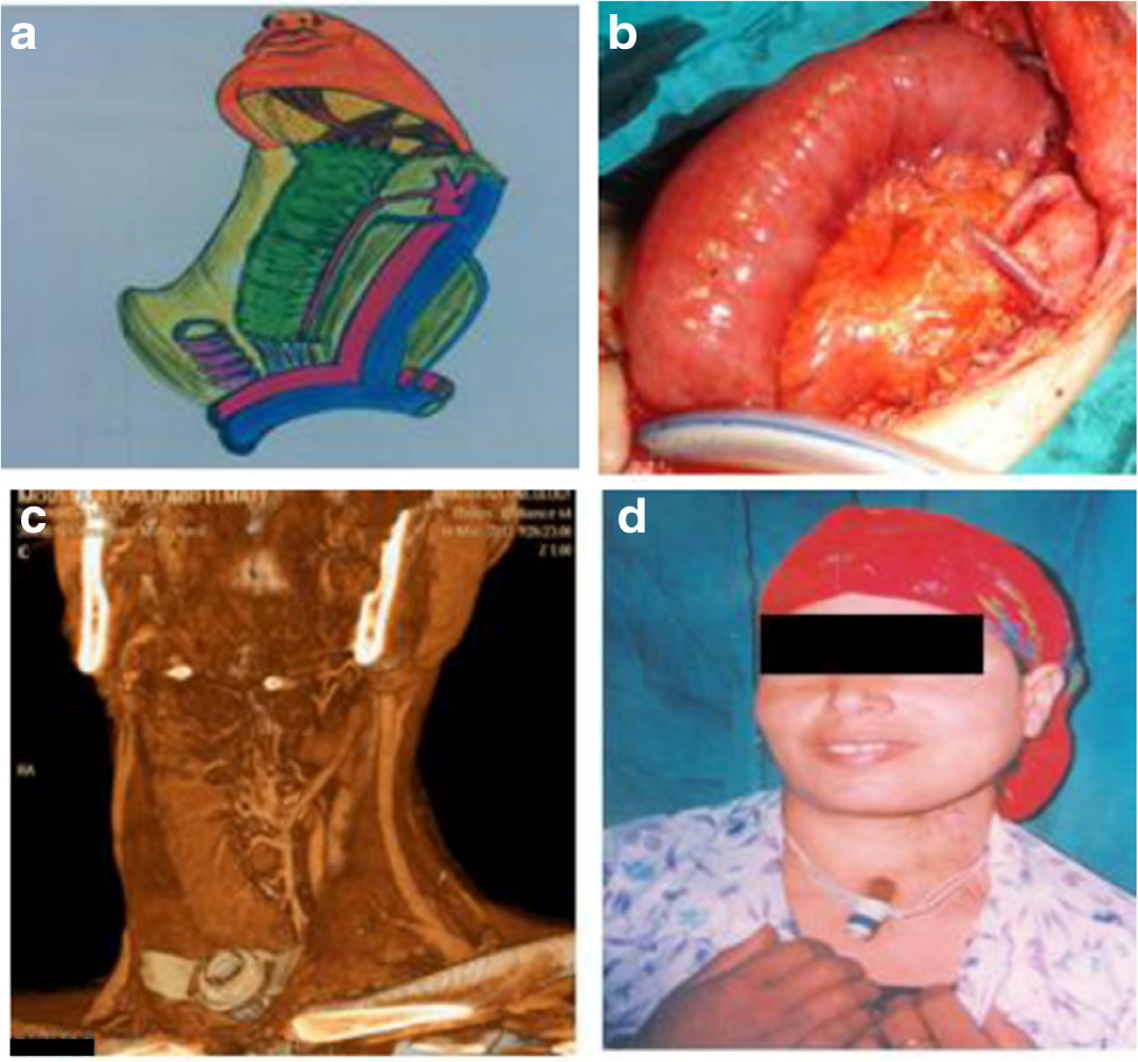
**Free jejunal flap. (a)** Diagrammatic scheme. **(b)** View of the flap after anastomosis. **(c)** Postoperative multislice computed tomography showing patency of the arcades after anastomosis. **(d)** Five-year postoperative view.

**Figure 5:**
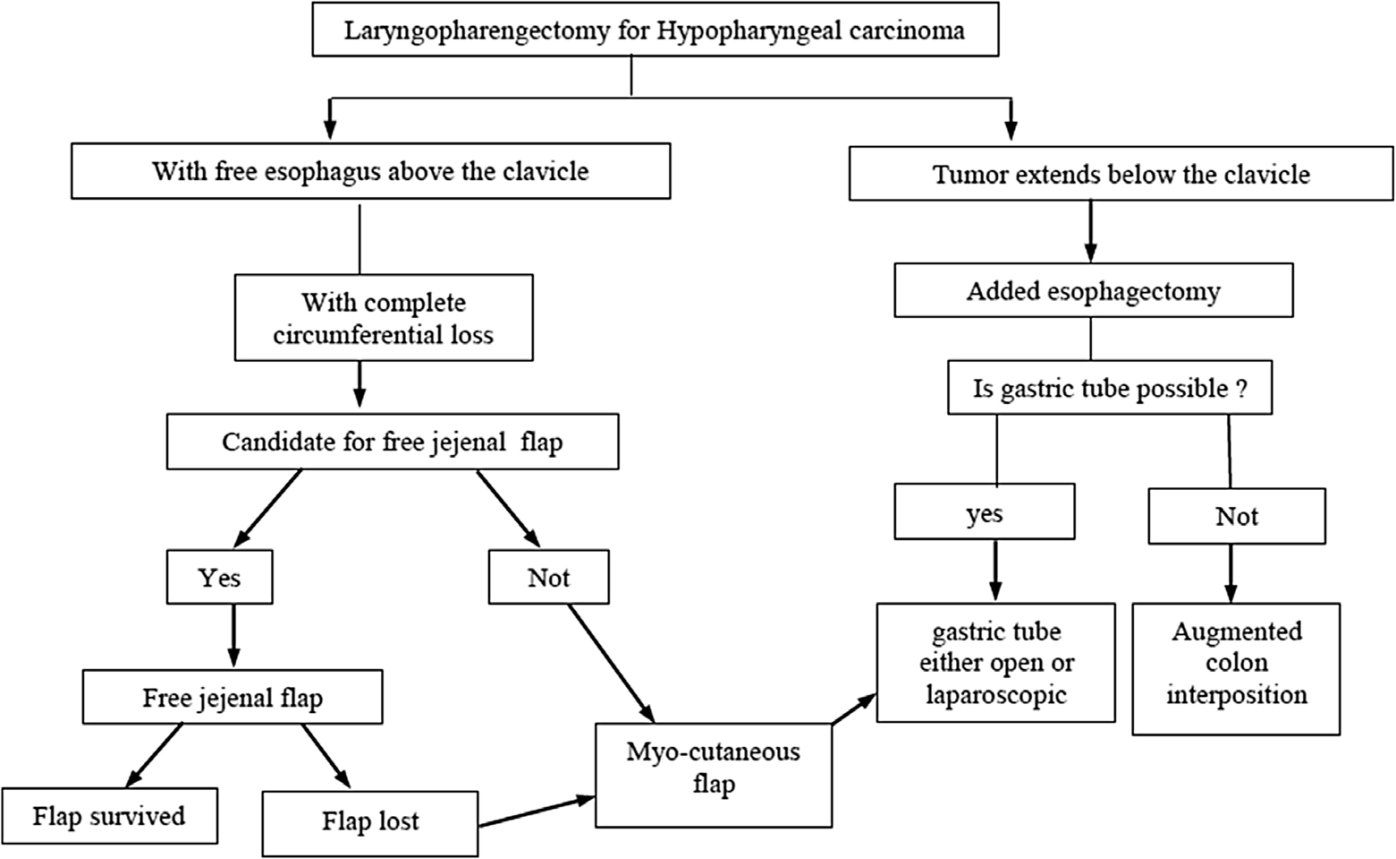
Algorithm for the management of defects after laryngopharyngectomy for hypopharyngeal carcinoma.

Reconstruction of the pharyngeal defect after total pharyngectomy for hypopharyngeal cancer is one of the most challenging fields of surgical oncology. Multiplicity of options usually means that none is ideal. Each option has its advantages and disadvantages. It is clear that morbidity and mortality increase if esophagectomy is added and the mediastinum is attacked. This is due to the increasing magnitude of the operation and the number of reconstruction tools needed that will increase the overall operative trauma. Triboulet and collegaues reported a study that involved 209 cases with a mortality rate of 4.8% and a morbidity rate of 38.3% [18]. Higher mortality rates were recorded in a study performed by Pesko and colleagues that involved 85 patients with 29 cases reconstructed by the stomach, 11 by colon bypass and 6 by free jejunal flap. They recorded a 13% mortality rate and a 50% morbidity rate [19]. Our mortality rate was 10.6% and morbidity rate was 31.7%. Regarding pharyngectomy without esophagectomy, in which the lower pharyngeal margin was free, the best reconstructive tool for function and low possibility of stricture was the free jejunal flap (Figure 4) (Table 3). Its main drawback is that it is more time consuming and more technically demanding than the myocutaneous flap. Similar results were obtained by others [5-10]. Recently the radial forearm flap and anterolateral thigh flaps were introduced [20], but these need further study before being compared to the free jejunal flap. In this study we can observe the relatively wider use of myocutaneous flaps, although their use carried the highest incidence of fistula and stricture with poorer functional outcome. The higher incidence of their use comes from their simplicity of harvest, which is more suitable in the relatively older age and those with associated poor tolerance to major operation. We recommended myocutaneous flaps to be reserved for coverage of partial defects, in those not candidates for free flap, or as a salvage in cases with free flap failure (Figure 5). In cases with infiltrated lower pharyngeal margin in which esophagectomy was indicated, although results of both gastric pull up and gastric tube are nearly similar as regard their good function and low complication rate, we prefer the gastric tube as it causes lower mediastinal compression and lower respiratory distress, thus decreasing respiratory complications (Figure 5). It could be done safely by laparoscopic technique with reduction of the abdominal wound complications. We reserve the colon bypass to cases not suitable for gastric tube in order not to perform major colectomy and colonic anastomosis. Finally we can say that the method of reconstruction does not affect survival or recurrence rate but it is important for better quality of life in such patients.

## Conclusion

We recommend free flaps as the free jejunal flap as a choice for reconstruction of circumferential pharyngeal defects as their use carries the best functional outcome with the lowest incidence of fistula and stricture, while the myocutaneous flaps are to be reserved for partial defects and cases who are not candidates for free flaps or as a salvage in case of free flap failure. With added esophagectomy we recommend the gastric tube as a first choice method (based on its better functional results and less incidence of early fistula) with the augmented colon being reserved for cases in which the stomach is not available or has failed.

## Consent

Written informed consent was obtained from the patient for the publication of this report and any accompanying images.

## Competing interests

The authors declare that they have no competing interests.

## Authors’ contributions

AD and MTH carried out the operations. AK, OH, SR, FS, WN, SK and KM participated in the collection of data and drafted and reviewed the manuscript. All authors read and approved the final manuscript.
